# Floating in Space: How to Treat the Weak Interaction between CO Molecules in Interstellar Ices

**DOI:** 10.1021/acsearthspacechem.3c00086

**Published:** 2023-06-14

**Authors:** Brian C. Ferrari, Germán Molpeceres, Johannes Kästner, Yuri Aikawa, Marc van Hemert, Jörg Meyer, Thanja Lamberts

**Affiliations:** † Leiden Institute of Chemistry, Leiden University, Leiden 2300 RA, The Netherlands; ‡ Department of Astronomy, Graduate School of Science, The University of Tokyo, Tokyo 113 0033, Japan; § Institute for Theoretical Chemistry, University of Stuttgart, 70569 Stuttgart, Germany; ∥ Leiden Observatory, Leiden University, P.O. Box 9513, 2300 RA Leiden, The Netherlands

**Keywords:** CO, binding energy, interstellar ices, astrochemistry, CO depletion, interstellar medium

## Abstract

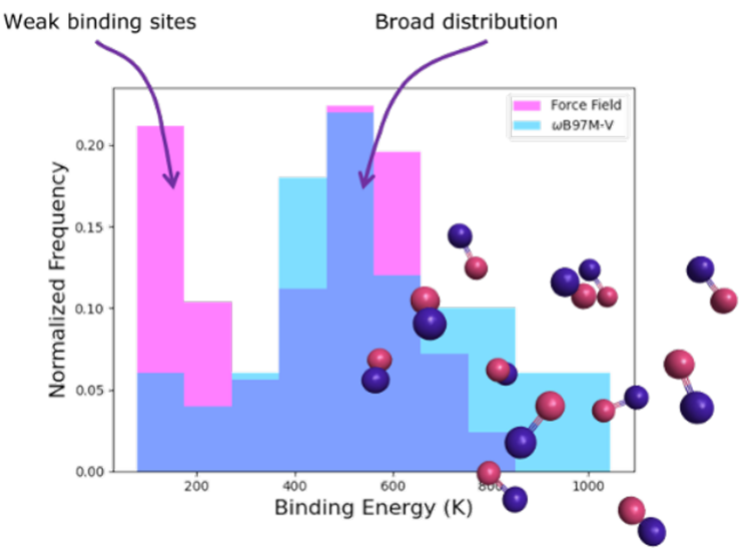

In the interstellar medium, six molecules have been conclusively detected in the solid state in interstellar ices, and a few dozen have been hypothesized and modeled to be present in the solid state as well. The icy mantles covering micrometer-sized dust grains are, in fact, thought to be at the core of complex molecule formation as a consequence of the local high density of molecules that are simultaneously adsorbed. From a structural perspective, the icy mantle is considered to be layered, with an amorphous water-rich inner layer surrounding the dust grain, covered by an amorphous CO-rich outer layer. Moreover, recent studies have suggested that the CO-rich layer might be crystalline and possibly even be segregated as a single crystal atop the ice mantle. If so, there are far-reaching consequences for the formation of more complex organic molecules, such as methanol and sugars, that use CO as a backbone. Validation of these claims requires further investigation, in particular on acquiring atomistic insight into surface processes, such as adsorption, diffusion, and reactivity on CO ices. Here, we present the first detailed computational study toward treating the weak interaction of (pure) CO ices. We provide a benchmark of the performance of various density functional theory methods in treating the binding of pure CO ices. Furthermore, we perform an atomistic and in-depth study of the binding energy of CO on amorphous and crystalline CO ices using a pair-potential-based force field. We find that CO adsorption is represented by a large distribution of binding energies (200–1600 K) on amorphous CO, including a significant amount of weak binding sites (<350 K). Increasing both the cluster size and the number of neighbors increases the mean of the observed binding energy distribution. Finally, we find that CO binding energies are dominated by dispersion and, as such, exchange-correlation functionals need to include a treatment of dispersion to accurately simulate surface processes on CO ices. In particular, we find the ωB97M-V functional to be a strong candidate for such simulations.

## 1 Introduction

Carbon monoxide (CO) is ubiquitous throughout the interstellar medium (ISM), and within molecular clouds it is one of the most abundant molecules. It was first detected in the gas phase in 1970^1^ and in the solid phase in 1979.^2^ Solid CO ice is typically identified by its features at 4.67 and 4.681 μm, which have been shown to correspond to CO embedded in apolar and polar environments, respectively.^3^ This aligns with the general idea that interstellar ices are layered, with first a polar, mixed, but water-rich inner layer and then an apolar, mixed, but CO-rich outer layer.^4,5^ This is supported by the novel James Webb Space Telescope (JWST) observation of NIR38, which found that fitted CO ice profiles are dominated by a pure component with two weaker mixed components.^6^

Recent studies claim that the CO-rich ice is formed as a single crystal atop an amorphous water-rich ice.^7^ Kouchi et al. showed that amorphous CO (am-CO) deposited on amorphous solid water (ASW) was highly susceptible to Ostwald ripening during crystallization, resulting in only a few crystal islands growing with others feeding into the larger ones.^7,8^ They also showed that both UV and electron irradiation of crystalline α-CO do not destroy the crystal structure. Complementary to this work, He et al. have found that pure CO ice under interstellar conditions and time scales is likely to be crystalline.^9^

During deposition, Kouchi et al.^7^ found that CO only partially wets the ASW surface, whereas Noble et al.^10^ found complete wetting. In the latter study, wetting was determined via temperature-programmed desorption (TPD), whereas the former directly imaged the surface during deposition with transmission electron microscopy (TEM). In TEM imaging, the thickness of the sample is determined by the darkness of the image. Identifying the growth of a single monolayer through this method is quite challenging. To reconcile the differences in observed wetting, Kouchi et al.^7^ evaluated inequalities based on binding energies to suggest that CO first completely wets the ASW surface and then partially wets the CO surface coating the ASW. One crucial assumption was that the binding energy for CO on α-CO is identical to the binding energy for CO on am-CO. It is important to emphasize that this particular assumption (*vide infra*) has not been verified by any experiments so far.

While the majority of studies on the binding energy of CO are experimental, they yield a wide range of values, due to the different empirically motivated pre-exponential factors used to fit the data from TPD curves.^11−14^ TPD studies have difficulties distinguishing the different binding on amorphous versus crystalline CO, because of (a) the narrow desorption temperature range and (b) the dependence on the experimental heating rate. Temperature interval desorption (TID) experiments can circumvent the dependence on the heating rate^14^ but are susceptible to deviations arising from restructuring. Thus, computational studies with an appropriate level of accuracy are pivotal to understand the binding energy of pure CO at an atomistic level.

Computational studies on CO binding energies have been sparse, because finding an appropriate balance between accuracy and computational cost is very challenging for this weakly bound system.^15,16^ High(er) levels of (*ab initio*) theory cannot describe systems large enough to mimic amorphous systems. Thus, to allow larger system sizes, other techniques, such as force-field-based classical molecular dynamics,^17,18^ continuous time random-walk Monte Carlo simulations,^19^ and kinetic Monte Carlo simulations^20,21^ have been applied in recent years.

Here, we report a benchmark of a previously parametrized^17^ classical force field (FF) against coupled cluster theory and a variety of exchange-correlation functionals rooted in density functional theory (DFT) with the aim of determining a reliable and cost-effective way to treat surface processes on CO ices. To this end, we compare binding energies for CO on am-CO which is modeled by small CO clusters. We further report FF-based results for the effect of cluster size on the binding energy distributions, as well as binding energies of CO on α-CO. Our results are placed in the astrochemical context and aim to lay the groundwork to provide recommendations for future selections of techniques and cluster sizes for studies of adsorption or dynamics on interstellar CO ices.

## 2 Computational Details

### 2.1 Force Field

The force field uses a site–site pair potential that was parametrized by van Hemert et al.^17^ based on CCSD(T) calculations for the CO dimer using the aug-cc-pVQZ basis set in combination with the Boys–Bernardi counterpoise correction. The force field has been reimplemented in Python in the form of an Atomic Simulation Environment (ASE) calculator.^22,23^ Our implementation of the force field used for the calculations in this work does not employ any cutoffs for energy and force evaluations. The values for all parameters are summarized in Table 1. We estimate the (intermolecular) many-body effects beyond pair interactions to amount to less than 2% of the total interaction energy (see Section 1 of the Supporting Information for details).

**Table 1:** CO–CO Potential Parameters

*V*_Morse_		
*r*_e_ (Å)	*D*_e_ (eV)	γ (Å^–1^)
1.1282	11.23	2.3281

	*V*_exch_ + *V*_disp_		
*i*, *j*	*A*_*ij*_ (eV)	*B*_*ij*_ (Å^–1^)	*C*_*ij*_ (eV Å^6^)
C, C	361.36	2.836	33.37
O, O	6370.10	4.253	10.52
C, O	1516.74	3.544	15.16

	*V*_el_	
*i*	*Q*_*i*_^0^ (e)	σ_*i*_ (Å^–1^)
C	–0.47	3.845
O	–0.615	2.132

The interaction potential is composed of an intramolecular Morse potential (*V*_Morse_) and intermolecular contributions that are intended to capture exchange (*V*_exch_), dispersion (*V*_disp_), and electrostatic (*V*_el_) interactions between pairs of CO molecules:
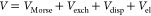
1The Morse potential is given by
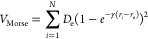
2where *D*_e_ is the dissociation energy, *r*_*i*_ is the CO bond length of the *i*th molecule, *r*_e_ is the equilibrium CO bond length, *N* is the number of molecules in the system, and γ is a constant. Both of the Morse potential parameters were obtained from fitting to experimental data. A Buckingham potential is used for the exchange and dispersion contributions:
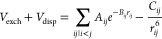
3Here, *r*_*ij*_ is the distance between the *i*th and *j*th atoms, and all other terms (*A*_*ij*_, *B*_*ij*_, *C*_*ij*_) are constants, given in Table 1. Note that *i* and *j* are atoms of different molecules. Finally, the electrostatic contribution is given by
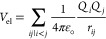
4where the point charges *Q*_*i*_ and *Q*_*j*_ are each located on a different molecule at a distance *r*_*ij*_ with respect to each other. The C and the O atoms form negative charge centers. A compensating total positive charge, *Q* = −(*Q*_C_ + *Q*_O_), is placed on the center of mass of each molecule, resulting in 9 Coulomb interactions between each pair of CO molecules. The charges mimic the *ab initio* derived dipole and quadrupole moments. The moments were originally calculated using MCSCF/CCI calculations with the aug-cc-pVQZ basis set, and the proportionality used for the charges is given by
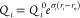
5where *r*_*i*_ is the intramolecular CO distance, *Q*_*i*_^0^ is the charge distribution at the equilibrium bond length, and σ_*i*_ is a constant, given in Table 1.

#### 2.1.1 Simulation Procedures

We generated the amorphous clusters through an in-house “hit-and-stick” script. A randomly oriented CO molecule was spawned 8 Å away from the surface of the CO cluster in a random direction with ∼50 K of translational energy in the direction of the center of mass of the CO cluster, followed by a 5 ps microcanonical (NVE) ensemble simulation using the ASE (version 3.22.1) implementation of the velocity Verlet algorithm.^24,25^ Subsequently, a geometry optimization was run with the ASE implementation of the Broyden–Fletcher–Goldfarb–Shanno (BFGS) optimizer^26^ using a convergence criterion of 1 × 10^–6^ eV/Å as a maximum force per atom. This procedure was repeated until the cluster reached a specified number of molecules which was passed to it as an input. Using this technique, 220 clusters were generated with sizes ranging from 8 to 350 CO molecules.

Binding sites for amorphous clusters were sampled by first constructing an α-shape,^27^ the “shape” of a set of finite points in space, around the atoms.^55^ The α parameter was optimized such that the α-shape would fully enclose all atoms while minimizing the volume enclosed. The vertices of the α-shape were then uniformly sampled and a new randomly orientated CO molecule was placed 3–5 Å away from the norm of the sampled vertex, with the restriction that no atom in the new molecule was closer than 3 Å from any cluster atom. This ensured that the binding sites selected were equally spaced both from each other and the nearest CO molecule. The cluster and admolecule complex was then optimized to a maximum force per atom of 1 × 10^–3^ eV/Å. The convergence criteria here and for the “hit-and-stick” procedure were carefully selected based on a study of their effects on the binding energy distributions (see Section 2 of the Supporting Information for details). After geometry optimization, the number of nearest neighbors was determined by counting all molecules with at least one atom within 3.6 Å distance of either C or O of the adsorbing CO molecule.

An α-CO crystal was created by using symmetry operators from the *P*2_1_3 space group with lattice parameters *a* = *b* = *c* = 5.9638 Å, α = β = γ = 90°. We first cut out a cubic (α-CO)_864_ finite cluster from this crystal, which was terminated by {100} faces, and optimized it with a tight (1 × 10^–6^ eV/Å) convergence criterion. To avoid edge effects except for where adsorption of additional CO molecules is being studied (i.e., at the “sides and at the bottom”), an (α-CO)_256_ cluster is cut out from the center of one of the (100) faces of the (α-CO)_864_ cluster. By freezing out the subsurface molecules they retain their crystalline character, and only the surface edges lose their crystalline character.

CO molecules were placed initially at distances of 3 Å above the crystal face. A total of 500 randomly sampled sites with a randomly orientated CO molecule were used to calculate the binding energies. Only the top layer and admolecule were free to move during optimization; the complex (CO + α-CO_256_) was optimized with the BFGS optimizer and a moderate (1 × 10^–3^ eV/Å) convergence criterion. A buffer region between the plane and crystal edge was left to ensure edge sites would not be included in the binding energy distributions. The above procedure aims for a realistic simulation of adsorption on a α-CO crystal considering periodic boundary conditions were not applied.

Binding energies for both am-CO and α-CO are calculated via
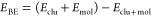
6where *E*_clu_ is the energy of the optimized cluster, *E*_mol_ is the energy of a single optimized CO molecule, and *E*_clu+mol_ is the energy of the optimized complex (cluster with adsorbed CO). Vibrational zero-point energies (ZPE) were calculated within the harmonic approximation for am-CO, and their contribution to the binding energy is given via ΔZPE = (ZPE_clu_ + ZPE_mol_) – ZPE_clu+mol_. Vibrational frequency calculations were carried out with the ASE (version 3.22.1) vibrations package, using finite displacements of ±1 × 10^–4^ Å. All systems studied in this work yielded a total of 4 or fewer imaginary modes, which were not related to the adsorbate motion. The sum of their absolute values corresponded to a ZPE of 0.2 meV or less, which was omitted when calculating the ZPE corrections.

### 2.2 Density Functional Theory

We compared the interaction energy (Δ*E*_int_ = *E*_dimer_ – 2*E*_CO_) of four randomly oriented CO–CO dimers optimized at the CCSD(T)/ma-def2-TZVP level of theory, with the ORCA program (version 5.0.3),^28,29^ against a series of exchange and correlation density functionals. Given the importance of the dispersion interaction for weakly interacting systems,^30^ we treated dispersion in two ways: (A) *via* Grimme’s dispersion correction, either the fourth generation D4^31^ or D3^32^ including three-body contributions and (B) including a fraction of nonlocal (NL) correlation energy in the exchange and correlation energy. Under the latter formalism, the total exchange and correlation energy becomes
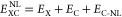
7where *E*_X_ and *E*_C_ denote exchange and correlation energies according to a particular functional, and *E*_C-NL_ accounts for nonlocal correlation following the formalism of Vydrov and Van Voorhis.^33^ All DFT calculations used the ma-def2-TZVP^34,35^ basis set, including a minimal set of diffuse functions to capture long-range interactions, and were carried out with the ORCA program (version 5.0.3).^28,29^ Due to a bug in ORCA version 5.0.3 associated with the D4 method, calculations using this method were corrected with ORCA version 5.0.4. All-but-one corrections were smaller than 10 K. To minimize numerical errors, a large integration grid (defgrid3) was used throughout the calculations. This is most important for meta-GGA functionals prone to bigger integration errors; however, to ensure consistency between calculations all functionals were integrated with the same numerical grid. For all functionals, except ωB97M-V, the calculations made use of the RIJK technique, e.g. resolution of the identity for the Coulomb and exchange integrals. For the ωB97M-V functional, a *chain of spheres* evaluation of exchange (RIJCOSX in ORCA) was employed.

Furthermore, we compared DFT-based binding energy distributions with FF-based distributions. For the DFT-based distributions, CO clusters with sizes of 8, 10, and 12 molecules have been generated as described previously by Molpeceres and Kästner.^36^ This procedure differs from the “hit-and-stick” procedure mentioned in Section 2.1.1, because such an individual sticking procedure carried out at the DFT level would be too computationally expensive for the cluster sizes under consideration. Instead, initial structures were randomly generated using Packmol.^37^ Subsequently, the structures were preconditioned using the generic GFN-FF method within the extended tight-binding (xTB) theoretical framework in the following way.^38,39^ A long molecular dynamics (MD) simulation was run at 100 K for 100 ps for each structural model, to generate different starting configurations, applying a spherical wall potential to confine the CO molecules within the cluster and prevent evaporation of the ice. From this, we extracted an MD snapshot every 20 ps, which was quenched to 10 K for an additional 10 ps. These five initial structures per cluster size (15 in total) were subsequently optimized at the ωB97M-V/ma-def2-TZVP level of theory under strict convergence criteria: Max(*E*_diff_) = 1.0 × 10^–7^ E_h_, Max(step) = 3.0 × 10^–4^ bohr, RMS(step) = 2.0 × 10^–4^ bohr, Max(*F*) = 7.5 × 10^–5^ E_h_/bohr, and RMS(*F*) = 5.0 × 10^–5^ E_h_/bohr.

Binding energy calculations were performed by placing additional CO molecules around the cluster, spanning a distorted Fibonacci lattice, with the center of mass of each additional CO molecule at a (minimum) distance of 3 Å plus the maximum value of the Cartesian (*x*, *y*, and *z*) coordinate of any of the CO molecules in the cluster. More details for the sampling procedure can be found in ref (36). To avoid surface restructuring, these initial configurations were relaxed with slightly less strict convergence criteria: Max(*E*_diff_) = 2.2 × 10^–7^ E_h_, Max(step) = 4.0 × 10^–4^ bohr, RMS(step) = 2.6 × 10^–4^ bohr, Max(*F*) = 1.0 × 10^–4^ E_h_/bohr, and RMS(*F*) = 6.6 × 10^–5^ E_h_/bohr. For the (CO)_12_ cluster, we fixed all the molecules present at a distance higher than 6.5 Å from the admolecule’s center of mass, to ease the convergence of the geometry optimization procedure. Binding energies were calculated using eq 6. We note that the aforementioned geometry optimization thresholds are less strict than those used for the FF calculations, owing to the fact that the energy and force calculations at the DFT level increase the computational time by about 5–6 orders of magnitude. Nevertheless, according to our detailed analysis in Section 2 in the Supporting Information, we do not expect this to have a significant effect on the calculated binding energies.

## 3 Results and Discussion

### 3.1 DFT Benchmark

**Figure 1:**
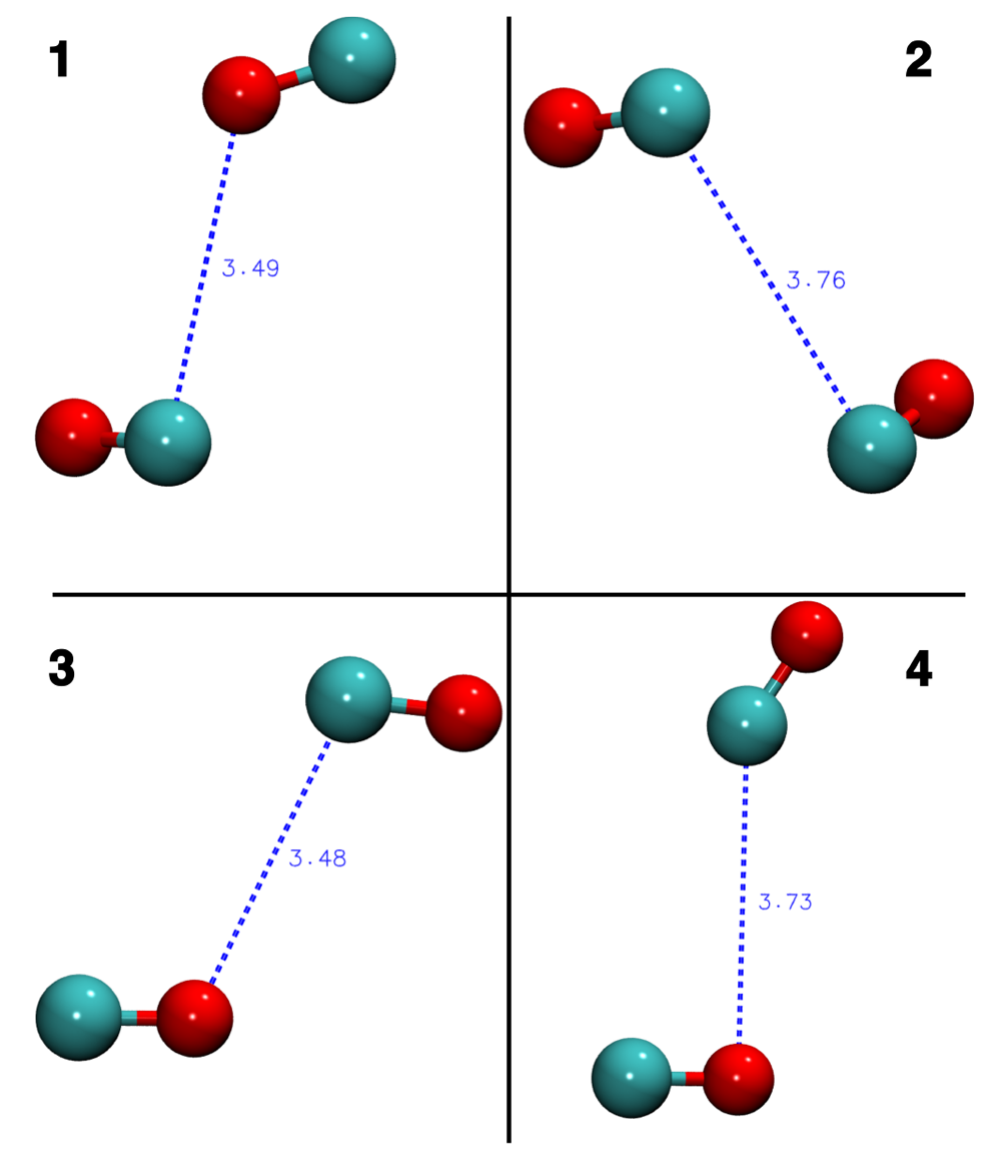
CO dimer configurations used to determine the interaction energies utilized in our benchmark study (Table 2). Full geometry coordinates are given in the Supporting Information.

The following functionals were tested: PBE-D3,^40^ B3LYP (without dispersion),^41^ B3LYP-D4,^31,41^ B3LYP-NL,^33,41^ REVPBE0-D4,^31,42^ REVPBE0-NL,^33,42^ BHLYP-D4,^31,43^ M06-2X-D3,^32,44^ PW6B95-D4,^31,45^ PW6B95-NL,^33,45^ and ωB97M-V,^46^ which has the NL treatment in its original implementation. For each of these functionals, the dimer structure was reoptimized starting from the CCSD(T)/ma-def2-TZVP optimized geometries. The structures of the four dimer configurations are depicted in Figure 1 (see Section 5 in the Supporting Information for coordinates), and the calculated interaction energies are summarized in Table 2.

**Table 2:** Dimer Interaction Energy, *E*_int_, for the Reference Method CCSD(T)/ma-def2-TZVP and the Difference Δ*E*_int_ = *E*_int,CCSD(T)_ – *E*_int,DFT_ for Different Exchange and Correlation Functionals in kelvin

		dimer 1	dimer 2	dimer 3	dimer 4
CCSD(T)	*E*_int_	*-158.4*	*-163.4*	*-149.3*	*-112.0*
					
PBE-D3	Δ*E*_int_	68.2	63.0	77.2	114.5
B3LYP		–153.2	–152.0	–143.1	–122.2
B3LYP-D4*		9.9	–18.4	12.2	3.6
B3LYP-NL		–15.2	–28.7	–0.2	–24.3
BHLYP-D4*		46.6	22.5	47.8	22.9
M06-2X-D3		–19.2	–9.4	–18.0	–5.4
PW6B95-D4*		69.8	69.7	69.1	88.7
PW6B95-NL		59.0	67.1	55.3	76.3
REVPBE0-D4*		48.3	26.3	52.8	58.2
REVPBE0-NL		24.3	21.6	32.8	36.1
ωB97M-V		**0.7**	**1.6**	**-0.3**	**4.8**

Overall, the degree of agreement among all DFT methods including dispersion and CCSD(T) is satisfactory, confirming previous calculations.^18,47,48^ We observe that B3LYP without dispersion correction yields results that are far from the reference value, while both D4 and NL corrections bring the values much closer to the reference. In general, NL-corrected functionals perform only slightly better compared to those with a D4 correction. Furthermore, PBE-D3, a commonly used method in computational solid-state chemistry, clearly does not capture the interaction well, while the ωB97M-V functional outperforms all others. We further scrutinized the performance of the ωB97M-V functional by expressing the dimer potential explicitly in terms of radial and angular variables, similar to the method for the force-field construction (see the Supporting Information for details). We found good agreement between the ωB97M-V/ma-def2-TZVP potential and the CCSD(T)/aug-cc-pVQZ potential. Finally, note that a direct comparison to the energetics for these dimers predicted by the force field is not straightforward, as a result of the use of different basis sets for the CCSD(T) calculations and the FF parametrization.

The ωB97M-V/ma-def2-TZVP level of theory will be used for all future DFT calculations discussed here, and the applicability of this method to general adsorbates and reactions on ice clusters will be the subject of future work.

### 3.2 Amorphous CO

#### 3.2.1 Comparison between FF and DFT

**Figure 2:**
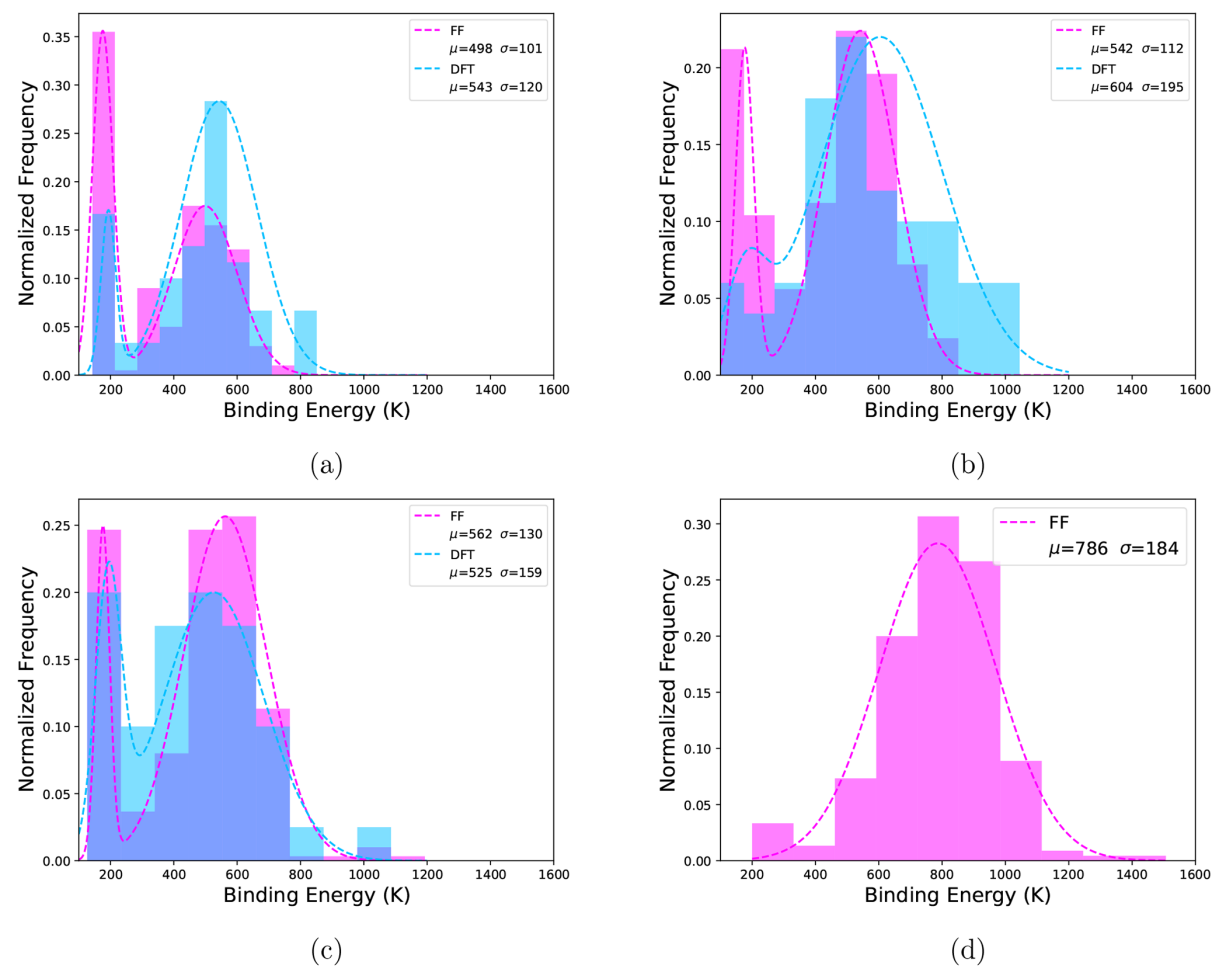
Distributions of binding energies calculated by DFT (blue) and FF (pink) methods for cluster sizes of (a) 8, (b) 10, (c) 12, and (d) 350 CO molecules. The mean (μ) and standard deviation (σ), both in K, for each distribution are shown in the plot legends. Dotted lines are probability density functions fitted to the binding energy distributions. For all distributions 10 equally spaced bins were used for plotting. Distributions consist of (a) 60 DFT samples and 200 FF samples, (b) 50 DFT samples and 250 FF samples, (c) 40 DFT samples and 300 FF samples, and (d) 550 FF samples. Note that overlapping blue and pink bars result in a third color within the plots.

We compared DFT- and FF-based binding energy distributions for cluster sizes of 8, 10, and 12 CO molecules. Thanks to the computational efficiency of the FF method, we have used larger sample sizes of 200, 250, and 300 respectively, in comparison to 60, 50, and 40 for the DFT calculations. The resulting distributions are depicted in Figure 2. Only for small clusters (16 or less CO molecules) were the distributions bimodal; as such, only those distributions were fit with two Gaussian profiles. In both Figure 2 and Table 3, for the bimodal cases only the descriptors for the higher BE distribution are shown. We find very good agreement between the DFT and FF binding energy distributions. The correspondence between FF and DFT results are underpinned by the descriptors of the distributions for all amorphous clusters studied, presented in Table 3. The standard deviations (σ, governing the width of the distribution) are in agreement even in cases where the means (μ, governing the average binding energy) differ between FF and DFT. However, the median (η) and the median absolute deviation (MAD), on the other hand, are more robust descriptors of the distributions. The median of the distribution is less sensitive to outliers and, in line with earlier work, we recommend using the median to estimate binding energies in these weakly bound systems.^18^

**Table 3:**
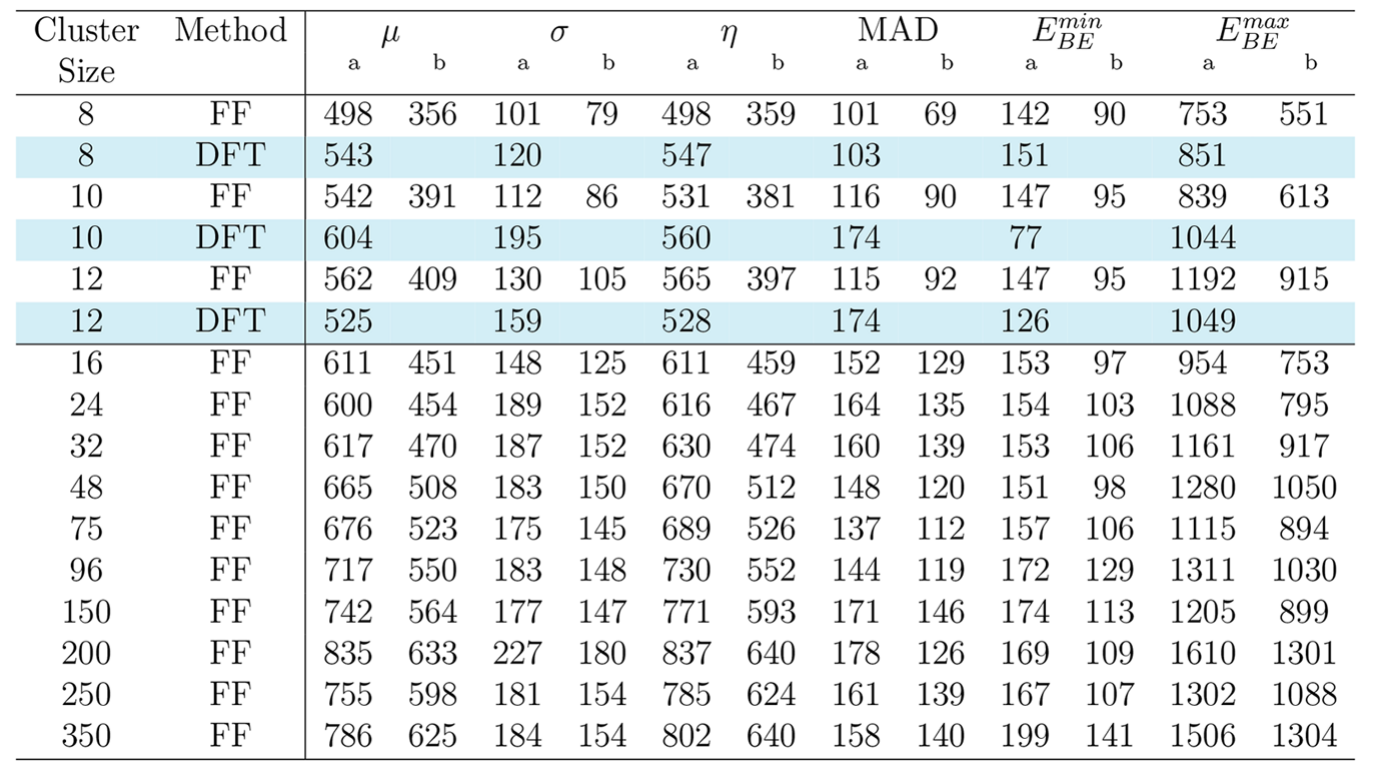
Mean (μ), Standard Deviation (σ), Median (η), Median Absolute Deviation (MAD), Smallest Binding Energy (*E*_BE_^min^), and Largest Binding Energy (*E*_BE_^max^) of the CO–COn^c^

a Without ZPE corrections.

b With ZPE corrections.

c All values in the table are in Kelvin.

Both methods find many weakly bound configurations, however, more so for the FF method. In particular, there is a significant amount of extremely weak (<200 K) binding sites. Subsequent NVE MD simulations at the FF level (with time steps of 0.5 fs) revealed that these binding sites are transient. Residual forces in those weakly bound configuration were sufficiently large so that the adsorbed CO diffuses to a stronger binding site during short time scales (∼1 ps). The relative abundance of these sites for FF simulations diminishes with increasing cluster size (see Figure 2d); as such, studies with small cluster sizes should scrutinize the relative abundance of these sites. For cluster sizes larger than 100 CO molecules these sites make 1% or less of the sample size, and for 350 CO molecules it is down to 1 transient site out of 550 samples. Since these sites disappear for larger cluster sizes, it implies they arise from adsorption at defect sites, where there is a reduced number of interactions.

#### 3.2.2 Dispersion Contribution

**Figure 3:**
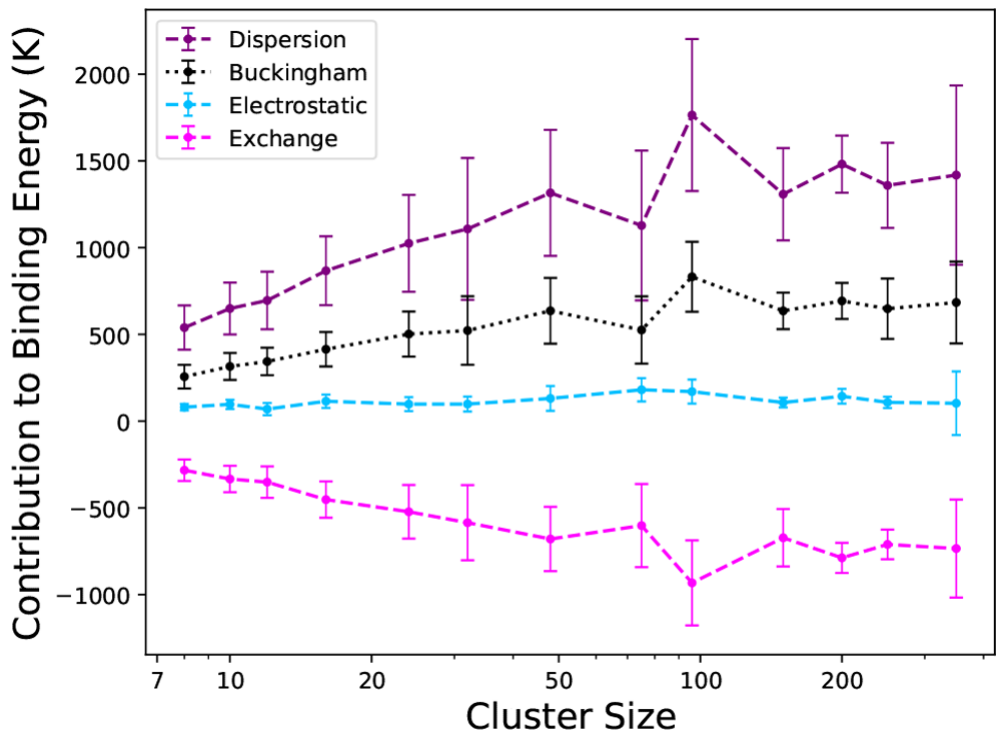
Average contribution to the binding energy of each part of the force field employed. Points are the mean of the contribution distributions, error bars are the standard deviations, and dashed/dotted lines are guides for the eye.

Figure 3 shows the contribution of each intermolecular interaction on the binding energy of CO on amorphous CO clusters. The dispersion (*V*_disp_) contribution dominates the binding energy, as expected, further underpinning the importance of the inclusion of dispersion in exchange-correlation functionals used to simulate surface processes on CO ices. We also include the Buckingham potential (*V*_exch_ + *V*_disp_) contribution since it illustrates the interplay between the exchange and dispersion contributions. Interestingly, the dispersion and exchange contributions vary significantly across binding arrangements, whereas their combined interaction (Buckingham) seems to balance each other out to produce a contribution with less variation. In contrast to water clusters, here the electrostatic (*V*_el_) contribution is the weakest (by absolute magnitude) contribution to the binding energy, with ∼100 K of the binding energy resulting from electrostatic interactions.

(CO)_*x*_ clusters have a key peculiarity in comparison with (H_2_O)_*x*_ clusters, which has been the most commonly used substance to simulate interstellar ices. In water, the dominant interaction is brought about by the hydrogen bonds between molecules, which is directional and strong. On the contrary, in the solid state CO ices most of the interaction energy stems from the dispersion interactions; as such, CO molecules tend to orient with less directionality than H_2_O. As a consequence, while dual-level calculations with electronic energy refinement on a low-level geometry are a cheap and accurate way to describe reaction energetics, the physisorption of CO admolecules on CO clusters is not well captured.

#### 3.2.3 Binding Energy Size Trend

**Figure 4:**
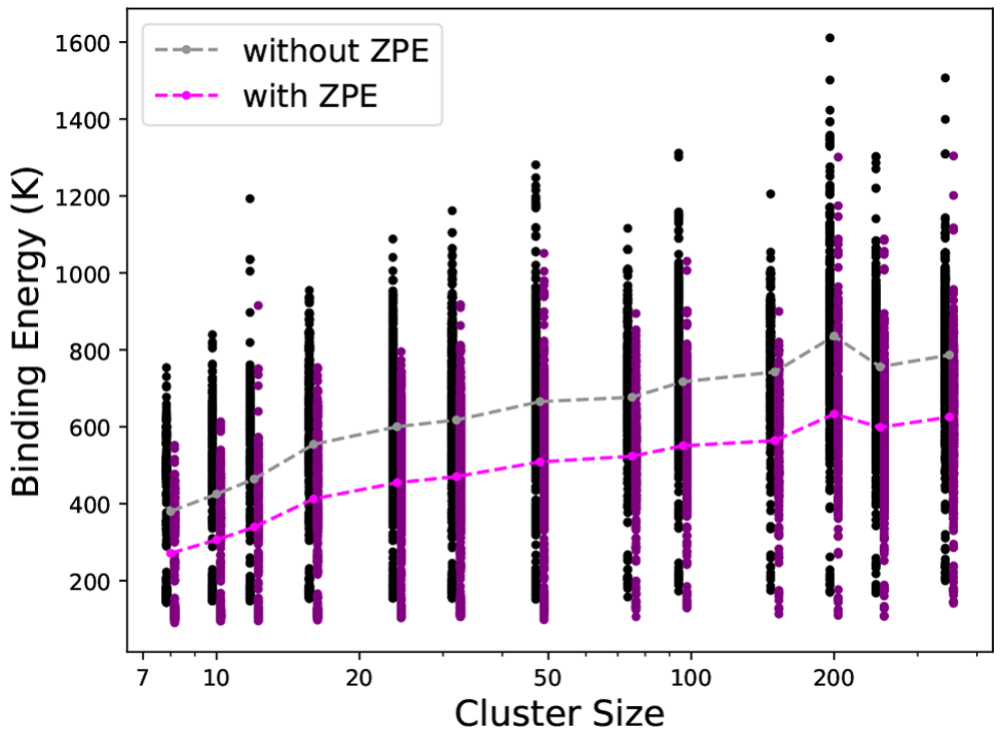
Black and purple dots are binding energy distributions, and the gray and fuchsia dots are the median values of the distributions. Dashed lines do not indicate predicted trends.

It is important to study the cluster size dependence because the weak dispersion results in short-range interactions that do not dominate over the long-range ones. We find that the median binding energy for a cluster size of 350 molecules (802 K) is nearly twice the median binding energy for a cluster size of 8 molecules (426 K). Figure 4 shows the effect of cluster size on the binding energy distribution, where both ZPE corrected and uncorrected binding energies are shown. The binding energies are shown as black and purple scatter points and demonstrate a wide range of binding sites on am-CO. Larger cluster sizes increase the overall binding energy, with the median value seeming to asymptotically approach the previously reported experimental values by Acharyya et al.^12^ (858 ± 15 K) and Bisschop et al.^13^ (855 ± 25 K).

**Figure 5:**
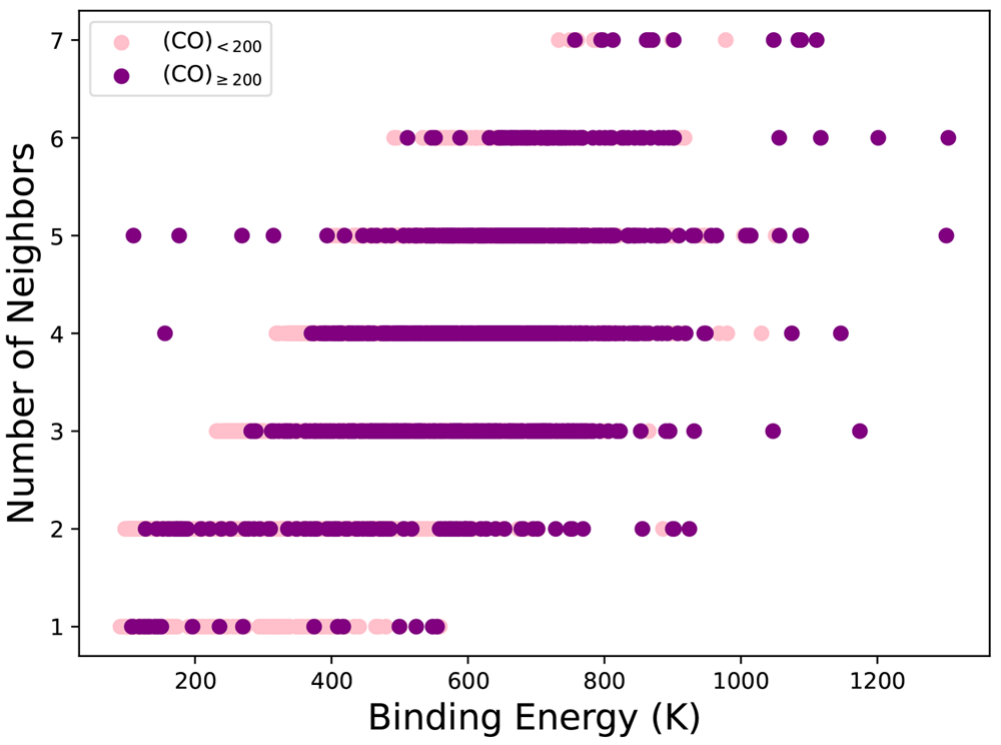
All FF calculated binding energies with ZPE corrections plotted as a function of number of nearest neighbors. Results for cluster sizes less than 200 molecules are shown in pink and all larger clusters are shown in purple.

In general, the largest binding energy at each cluster size increases with increasing size until 50 CO molecules. The (CO)_12_ cluster is an exception due to one of the (CO)_13_ complexes (CO + (CO)_12_) having a uniquely symmetrical orientation. This allowed the adsorbing CO to maximize its number of nearest neighbors, producing a binding energy significantly higher than on most other clusters. Larger clusters have maximum binding energies that depend less on the cluster size and depend more on the surface morphology and the number of nearest neighbors. Figure 5 shows the relationship between binding energy and number of nearest neighbors. Increasing the number of neighbors shifts the distribution of binding energies to larger values, because short-range interactions contribute more to the binding energy.

The ZPE correction lowers the median binding energy by 20–30% and reduces the MAD by 15–20%. This reduction is greater than or equal to ZPE contributions found from previously reported studies of other molecules on water ice surfaces.^48,49^ For a weakly interacting system, such as CO on CO, the ZPE corrections can influence binding significantly and should be carefully considered. The ZPE corrections are calculated within the harmonic approximation. This may result in an overestimation, because the weak CO–CO interactions on a relatively flat PES are expected to be dominated by anharmonic character. We find that the low-frequency modes dominate the ΔZPE with the largest contributions resulting from the frozen-out rotations and translation of the “newly” adsorbed CO molecule. Since these modes are more anharmonic in nature, we expect anharmonic corrections to the ZPE to be significant. As such, the ZPE corrections reported here are an upper limit on the exact ZPE correction.

### 3.3 Crystalline CO

**Figure 6:**
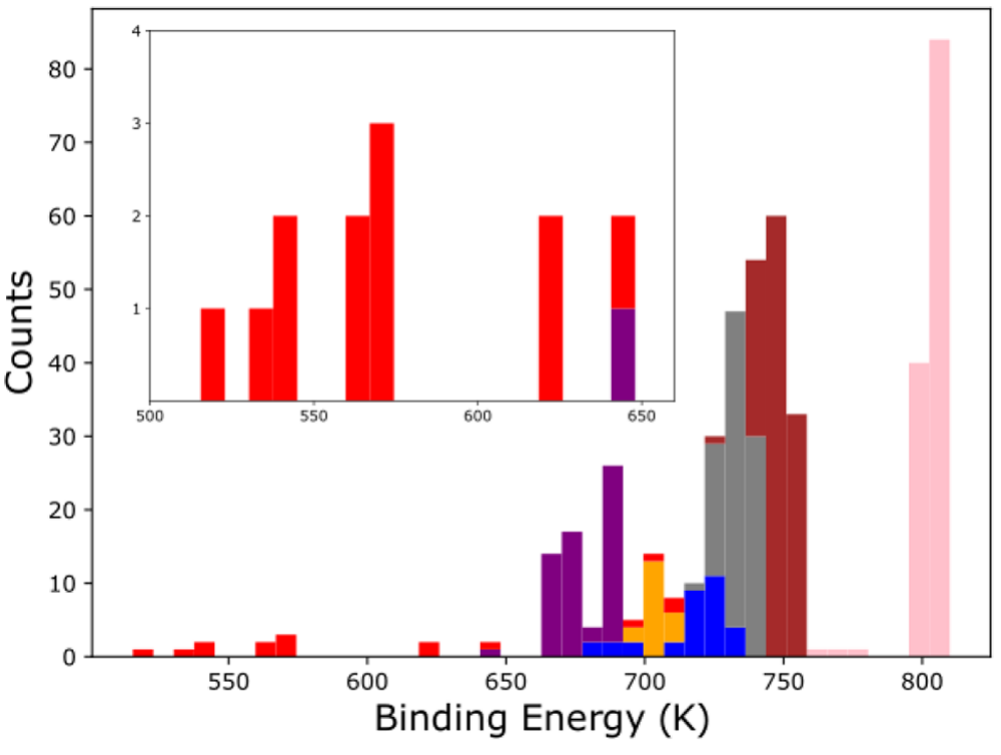
Distribution of binding energies calculated for a CO molecule adsorbed on the (100) surface of an α-CO crystal. Colors depict categories (see Figure 7) found by a clustering algorithm with reference to the orientation of the adsorbed CO. The inset is an enlargement of the low energy sites.

The binding energy distribution for CO on the (100) surface of α-CO is shown in Figure 6. The weakest binding sites (∼500 K shown in red in Figure 6) correspond to sites directly on top of a CO molecule on the topmost CO layer, and the strongest binding sites (∼800 K shown in pink in Figure 6) correspond to CO alignment with a subsurface α-CO molecule. Similar to the amorphous case, the weak binding sites (<650 K) are found to be transient, with CO diffusing into a site above a subsurface α-CO molecule when subsequent NVE simulations are performed. It then binds in orientations similar to those of the stronger binding sites mentioned above. Our calculations only consider the perfectly flat (100) CO crystal surface and thus do not include the influence of step edges. Ignoring these effects, we find that the median binding energy of CO on α-(CO)_256_ (742 K) is similar to that of CO on am-(CO)_250_ (785 K), validating assumptions made in previous studies.^7^

**Figure 7:**
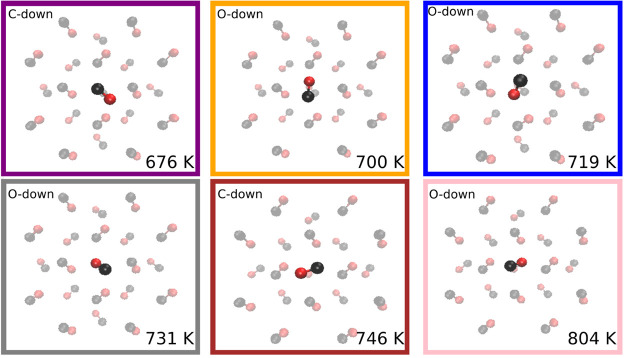
Representative image of each category for binding sites on the (100) surface of an α-CO crystal. Colored boxes indicate the category color in the histogram (see Figure 6), and text in the upper left corner of each box indicates which atom is closest to the crystal face. Text in the lower right corner of each box is the median binding energy of the categories distribution. “Noise” points (shown in red in Figure 6) are not shown here, since all orientations in the distribution differ significantly from each other. Note that all molecules shown here are adsorbed on a hollow site (over a CO molecule in the second layer).

In order to asses how the orientation of the adsorbing CO affects the binding energy, we ran a density-based spatial clustering of applications with noise (DBSCAN)^50^ algorithm on the orientation of adsorbed molecules. The molecules were assigned a category based on their relative alignment with the second crystal layer; the categories were then used to color the distribution in Figure 6 (see the Supporting Information for more details). A representative image for each category corresponding to the strongest binding sites is shown in Figure 7. The DBSCAN algorithm also separates “noise”, points that are not similar to other points based on clustering space, represented by the red bars in Figure 6. In general, we can conclude that the more parallel the orientation of the adsorbed CO is with the subsurface α-CO below it, the higher the binding energy.

Many binding sites center around 740 K, and they differ geometrically from the stronger sites around 800 K by the orientation of the adsorbed CO molecule (see Figure 7). Stronger binding occurs when the adsorbed molecule is approximately parallel to the subsurface CO, with the adsorbed CO being inverted (in terms of C-down or O-down). Although being the strongest binding orientation, this does not correspond to full alignment with the crystal structure. Those orientations have a CO molecule parallel to the subsurface CO, but noninverted and the binding energies fall within the distribution centered around 740 K.

## 4 Astrophysical Implications

We find that for large clusters (>200 CO molecules) roughly 10% of the binding energy distributions falls below 600 K. This supports the idea that CO molecules are mobile even at low temperatures. This could promote the crystallization of the CO ice on interstellar time scales, which would support recent claims of interstellar CO being in crystalline form.^7,9^ Whether or not the structural phase of CO ices has an effect on subsequent reactivity remains to be tested. It may lead to less accessible transition states for, e.g., the H + CO reaction, but given its intrinsic weakly interacting nature, it is possible that these effects are only observed in a single crystal without step edges or defects. As such, future studies on how the CO ice phase affects surface reactions would be of great interest to the astrochemical community.

The broad binding energy distributions reported herein are of particular interest to astrochemists modeling CO in prestellar cores. Within these regions there is evidence of a significant CO depletion in the gas phase; between 74% and 94% of the gas-phase CO is observed to be frozen out.^51^ However, models are unable to reproduce the observed gas-phase CO abundances and overestimate the CO depletion. For instance, Keto and Caselli were only able to reproduce the observed value by increasing the cosmic-ray induced desorption rate for CO by a factor of 30 from what is expected.^52,53^ Alternatively, Cazaux et al. showed that incorporating lower binding energy values for CO in models would decrease the CO depletion.^54^ They found that using a binding energy of 350 K would lower the CO depletion by 10% and a value of 300 K would lower it by 100%.^54^ Our results show that these binding energy values are within the distribution for CO. Additionally, we found that adsorption to defect sites is transient and in the limit of low CO coverage they will diffuse to stronger binding sites. At higher CO coverage, stronger binding sites will already be occupied and the admolecule will be unable to diffuse to a new site, resulting in a low-temperature desorption event. Incorporating these concepts along with their probabilities into models could result in gas-phase CO abundances closer to those observed, without the need to invoke additional processes.

## 5 Conclusion

In summary, we find that CO binding is dominated by dispersion and that many-body effects contribute minimally (<2%) to the interactions in CO ices. We also show that CO binding on amorphous CO occurs with a large range of binding energies (200–1600 K) and depends on both the cluster size and the number of nearest neighbors. Our results show a median binding energy of 802 K for (CO)_350_, which is nearly twice as large as that for (CO)_8_ (426 K). Accounting for ZPE within the harmonic approximation lowers the average binding energies by 20–30%. We expect this to be an upper limit because frustrated rotations and translations dominate the ZPE correction and the effect of these low-frequency modes might be overestimated by neglecting anharmonicity. This should be revisited in future studies.

We also report binding energy distributions (650–800 K) for CO binding on a flat α-CO crystal face, which we find to be orientation dependent. It also has a median value similar to that of amorphous CO, corroborating previous studies which assumed them to be equal. Lastly, we presented many dispersion-corrected functionals that performed well; however, we find the ωB97M-V functional performs the best when treating the CO–CO dimer interaction and suggest this functional to be used for general adsorbates and reactions on ice clusters.

## Supporting Information Available

Pair interaction validation, preliminary studies on the convergence criteria for FF-based calculations, spherical expansion analysis of the ωB97M-V functional, explanation of the DBSCAN clustering procedure, and Cartesian coordinates of dimer geometries (PDF)
