# Supplementary material for: Floating in Space: How to Treat the Weak Interaction between CO Molecules in Interstellar Ices
Source: ACS Earth Space Chem. 2023 Jun 14;7(7):1423–32. doi: 10.1021/acsearthspacechem.3c00086 (PMC10364131; doi:10.1021/acsearthspacechem.3c00086)
Supplement: Supplementary file 1 — sp3c00086_si_001.pdf [file sp3c00086_si_001.pdf]

Supporting Information

Floating in Space: How to Treat the Weak Interaction between CO Molecules in Interstellar Ices

Brian C. Ferrari,† Germán Molpeceres,‡ Johannes Kästner,¶ Yuri Aikawa,‡ Marc van Hemert,† Jörg Meyer,† and Thanja Lamberts*,†,§

†*Leiden Institute of Chemistry, Leiden University, Leiden 2300 RA, The Netherlands*

‡*Department of Astronomy, Graduate School of Science, The University of Tokyo, Tokyo 113 0033, Japan*

¶*Institute for Theoretical Chemistry, University of Stuttgart, 70569 Stuttgart, Germany*

§*Leiden Observatory, Leiden University, P.O. Box 9513, 2300 RA Leiden, The Netherlands*

E-mail: a.l.m.lamberts@lic.leidenuniv.nl

1 Pair Interaction Validation

We estimate the (intermolecular) many-body effects beyond pair interactions to amount to less than 2% based on the procedure outlined below. We selected 20 trimer geometries and have performed CCSD(T)/aug-cc-pVQZ calculations using the same computational setup as used for the construction of the force field. Ten geometries were selected from the bulk of an unequilibrated $(CO)_{522}$ 'crystal' cluster, and the other ten were selected from the bulk of a $(CO)_{800}$ amorphous cluster equilibrated to 10 K.[1] For each geometry the total energy

was calculated (E_{total}) and the energies of each pair ($E_{dimer,i}$) with the difference (Eqn. 1) attributed to non-additivity.

$$\Delta E = E_{\text{total}} - \sum_i E_{\text{dimer},i} \tag{1}$$

We find that ΔE can be both positive or negative and varies between 0 and 2% of E_{trimer}, indicating that a pair potential approach to study, e.g., binding of pure CO ices, is justified.

2 Geometry Optimization Threshold

Using the force field employed in thie work, we investigated the convergence threshold criteria for the creation of interstellar relevant CO clusters and for the subsequent binding energy calculations and concomitant distributions. While global sampling schemes provide a systematic way to obtain meta-stable states, instead, here we use a more pragmatic approach. This is also of more practical relevance to electronic structure calculations given their much higher computational cost. To this end, two $(CO)_{32}$ clusters were generated with the hit-and-stick method discussed in the manuscript. We tested six convergence criteria for the residual forces, namely 1×10^{-x} with $x = 1, 2, 3, 4, 5, 6$ eV/Å, leading to a total of 12 generated clusters. Subsequently, for each cluster geometry optimizations were performed for 60 binding sites and binding energies were computed for each convergence criterion equal to or looser than the one used for cluster generation, leading to 2520 simulations. The binding energies are calculated in the same method as described in the main article. The results are depicted in Fig. S1. As to be expected, the effect of the convergence criteria can be rationalized as follows: (a) during cluster generation, tightening the criteria results in a less disordered cluster and (b) during adsorption simulations, tightening the criteria forces the adsorbing molecule to overcome small barriers and progress toward stronger binding sites.

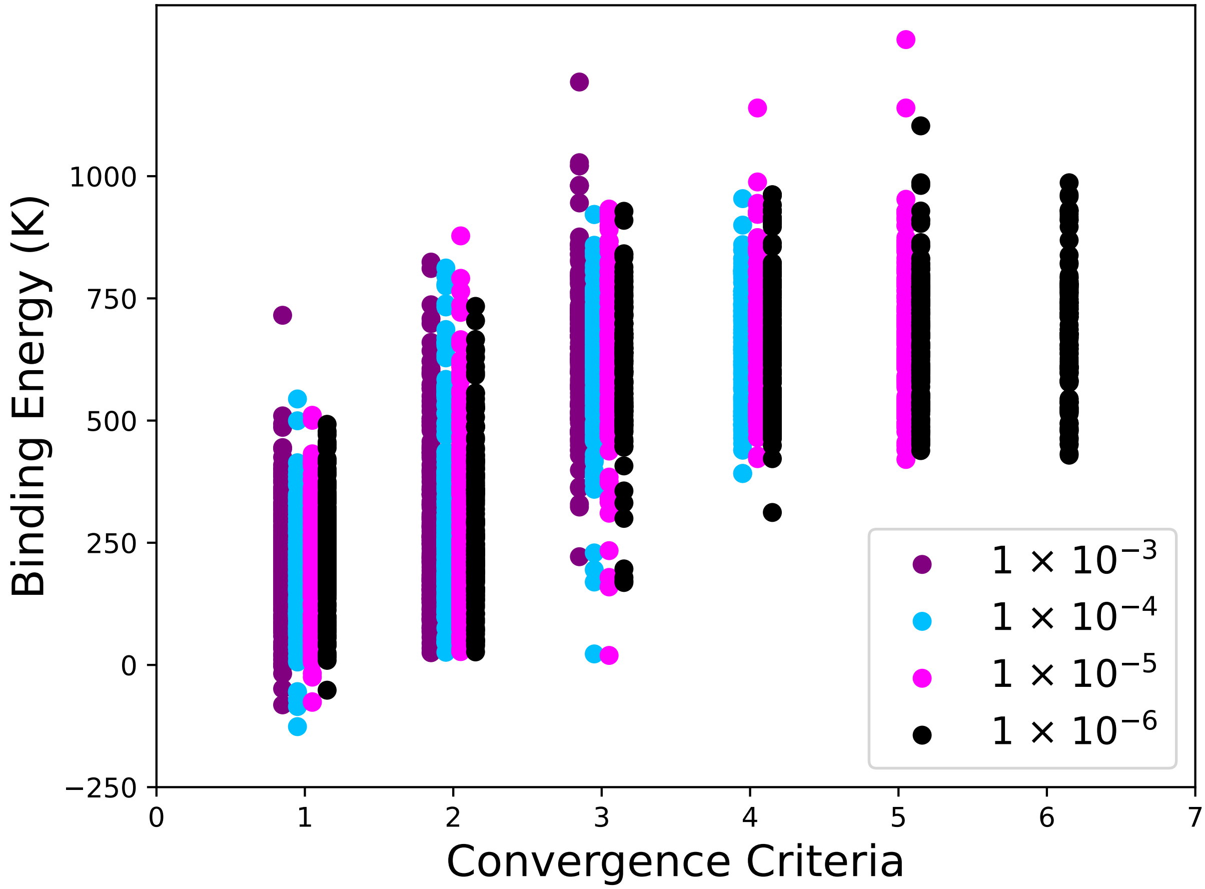

Figure S 1: Binding energy distributions for simulations with varying optimization criteria, note that the points are artificially offset on x-axis for visibility. The x-axis denotes the convergence criteria for the binding energy (adsorption) simulations based on 1×10^{-x} eV/Å. The colors indicate the thresholds used for the cluster generation: (a) purple dots used 1×10^{-3} eV/Å (b) cyan dots used 1×10^{-4} eV/Å (c) magenta dots used 1×10^{-5} eV/Å (d) black dots used 1×10^{-6} eV/Å.

Tightening the criteria for the cluster creation overall leads to narrower binding energy distributions which can be seen by comparing within each set of calculations per criterion on the x-axis in Fig. S1. For instance, for a threshold of 1×10^{-3} eV/Å on the adsorption simulations, the width of the black distribution (a threshold of 1×10^{-6} eV/Å for the cluster generation) is the smallest of all four colors. Tightening the criteria for the binding energy, *i.e.*, adsorption simulations, leads to an overall increase in binding energies and drastic reduction in the number of weak binding energies, because shallow minima will progress

toward more stable local minima.

Computational chemistry studies usually focus on well-converged structures corresponding to local minima, if not even the global minimum. In this case it would mean to make use of threshold criteria of 1×10^{-6} eV/Å for both cluster formation and adsorption simulations. However, for the study of binding on cryogenic (10 K) interstellar ices, this would represent an unrealistic scenario: adsorbing molecules have near to no additional energy to reorient on the surface. In particular, transient binding sites for which the binding energy is sufficient to lead to adsorption, yet so low that subsequent diffusion leading to chemistry or fast desorption is expected, can not be captured with stringent convergence criteria. In fact, for CO, it has been shown that these weakly bound sites determine the gas-phase depletion rates and influence astronomical models.[2] At the same time, loose convergence criteria leads to negative binding energies representative of a repulsive force. Thus, for our work, keeping the interstellar conditions and questions in mind, we use 1×10^{-6} eV/Å as a criterion for generating the clusters and 1×10^{-3} eV/Å for computing the binding energies at the force field level. As detailed in the manuscript, different criteria are used for the electronic structure calculations in light of the concomitant computational costs.

3 Total Interaction Energy

In order to further elucidate the virtues and shortcomings of the ωB97M-V functional, we applied a spherical expansion analysis[3,4] previously used by van Hemert et al. for the original construction of the force field. We label the intermolecular distance (the distance between centers of mass of the two molecules) by R and use dimer jacobi angles (θ_A, θ_B and ϕ) for the orientation. The potential is then expressed as,

$$V(R, \theta_A, \theta_B, \phi) = \sum_{L_a, L_b, L} c_{L_a,L_b,L}(R)\ A_{L_a,L_b,L}(\theta_A, \theta_B, \phi) \tag{2}$$

with R-dependent expansion coefficients c (in energy units), dimensionless basis functions A and the angular momentum coupling labels given by L_a,L_b and L.

We focus here on the two best performing exchange-correlation functionals, ωB97M-V and B3LYP-D4 both including dispersion corrections, along with bare B3LYP to understand better the effects of dispersion. All functionals employed the ma-def2-TZVP basis set, and CCSD(T)/aug-cc-pVQZ acts as the reference. We use the Molpro program for all methods except ωB97M-V, which was run with ORCA. For 21 different intermolecular distances (R), the energy of 294 orientations was calculated. In Figure S2 we present the intermolecular distance dependence of the dominant coefficients of the truncated expansion including 140 terms.

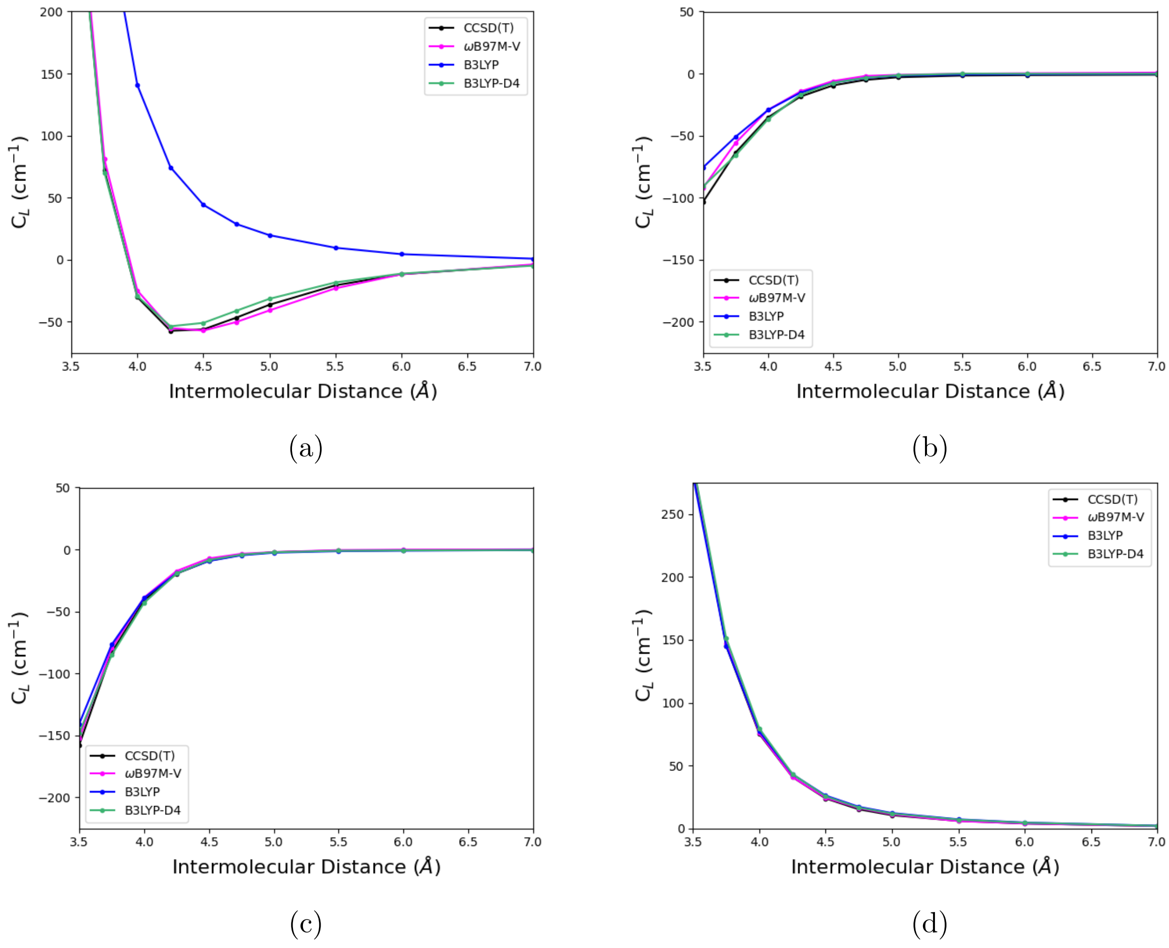

Figure S 2: The intermolecular distance dependence of the dominant coefficients of the 140 term expansion. Shown here are (a) c_{000}, (b) c_{112}, (c) c_{123}, and (d) c_{224}.

Figures S2 (b), (c), and (d) show the 3 dominant anisotropy terms. At long range they determine the orientational dependence of (b) the dipole-dipole interaction, (c) the dipole-quadrupole interaction and (d) the quadrupole-quadrupole interaction. For R values larger than 10 Å, they show the expected R^{-n}, with n = 3, 4, and 5 for case (b), (c) and (d) respectively.

For the isotropic component, Figure S2 (a), the CCSD(T) reference curve (black) has its minimum at R=4.25 Å with -50 cm^{-1} energy. Both B3LYP-D4 (green) and ωB97M-V (pink) reproduce the CCSD(T) curve closely, implying they incorporate dispersion in a similar way as CCSD(T). Bare B3LYP (blue) on the other hand demonstrates a lack of dispersion interaction, making it clear that B3LYP will always produce dimer equilibrium geometries with overestimated intermolecular distances.

This further underpins that ωB97M-V is well suited to treat CO ices, and may be able to describe other dispersion dominated systems as well. Considering its quality compared to its computational cost, we propose it to be an excellent functional for treating CO-CO interactions both for geometries and energies.

4 DBSCAN

Our goal in clustering the adsorbing geometries is to correlate the binding energy distributions with the orientation of the admolecule. In order to do so, we need to carefully select the features that will be supplied to the DBSCAN algorithm[5] to cluster the data. Within the α-CO crystal structure, there are two orientations of CO molecules per layer along the (100) direction, which defined the surfaces studied in the main article. Incidentally, the two have bond vectors that are nearly orthogonal. The relative alignment between the admolecule and one of these two CO orientations can be determined by taking the scalar product of unit vectors along the orientation of the two molecules. This provides a vector of 2 features that represents the general orientation of the admolecule, each in a range from -1 to

1. Figure 3 (a) shows these relative orientation features plotted for all sampled geometries prior to geometry optimization, and Figure S3 (b) shows them after geometry optimization. We then clustered the binding geometries based on these two features, using the DBSCAN algorithm. Figure S3 (c) shows the post-geometry optimization features plotted with color coded groupings determined by the DBSCAN algorithm.

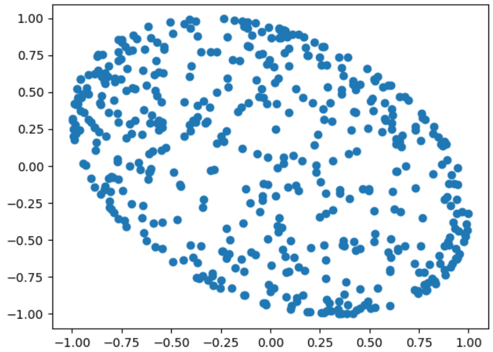

(a)

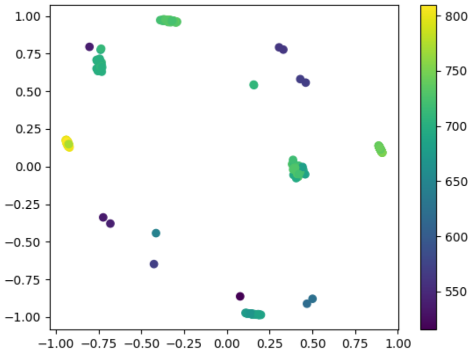

(b)

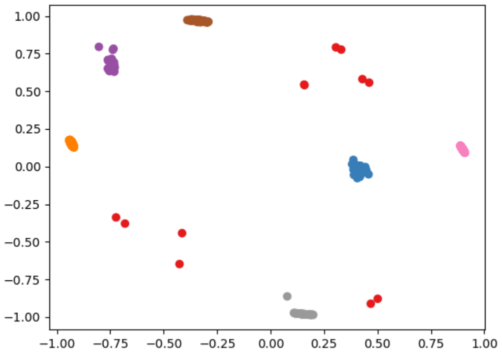

(c)

Figure S 3: Relative orientation features plotted, with $x-$ and $y-$ describing the relative alignment between the admolecule and the CO molecules in the second layer as described in the text. The plots show (a) all sampled geometries prior to optimizations, (b) all optimized geometries with color bar indicating the binding energy and (c) all optimized geometries after DBSCAN clustering where the colors indicate their grouping.

The DBSCAN algorithm requires the selection of two important parameters, ϵ which is the 'neighborhood' radius and N which is the minimum number of points within a 'neighborhood' needed to make a point a core point. In our study we varied both parameters

across 0.05 – 0.15 for ϵ and 3 – 6 for N, there was little difference in the resulting clusters. This is due to the tight packing of the data points. As such, we used a small value for the 'neighborhood' radius ($\epsilon = 0.05$) and a moderate minimum number of points ($N = 5$). This ensured that noise points were properly accounted for within the clustering scheme.

5 Dimer Geometries

Table S 1: Cartesian coordinates of dimer geometries.

Dimer 1	x	y	z
C	-0.19045519661532	-0.16135156541274	-0.90552295962114
O	-0.24781144304162	-0.21210633747172	0.22651845170074
C	2.05554035880910	1.76473141430682	1.93645844013598
O	1.92163442084784	1.61431360857763	3.05282589778442
Dimer 2	x	y	z
C	-0.02861196368715	0.14226748736038	-0.25438691023046
O	0.02917690189452	0.20456101059696	0.87674186711502
C	-0.05833331699430	-1.15110586453745	-3.94238709981301
O	0.00624938878693	-2.06114797341989	-3.26806560707154
Dimer 3	x	y	z
C	0.33269802910679	-0.27970796365053	-0.42781900490899
O	0.22589212548614	-0.11896432978238	0.69006372841763
C	-2.77905130484776	1.47739207659477	-2.53763134041904
O	-2.08999886745526	1.87256599749566	-1.72746787668853
Dimer 4	x	y	z
C	-0.46825007893992	0.20316684833911	-0.60019754337303
O	-0.47660149193020	0.25745548182980	0.53294588147191
C	3.05170157807967	-1.08533323930954	-0.35691671835964
O	3.01979134766368	-2.21624085850747	-0.43999546289307

References

(1) van Hemert, M. C.; Takahashi, J.; van Dishoeck, E. F. Molecular Dynamics Study of the Photodesorption of CO Ice. *Journal of Physical Chemistry A* **2015**, *119*, 6354–6369.

(2) Cazaux, S.; Martín-Doménech, R.; Chen, Y.; Caro, G. M.; Díaz, C. G. CO Depletion: A microscopic perspective. *The Astrophysical Journal* **2017**, *849*, 80.

(3) Berns, R. M.; van der Avoird, A. N_2–N_2 interaction potential from abinitio calculations, with application to the structure of $(N_2)_2$. *The Journal of Chemical Physics* **1980**, *72*, 6107–6116.

(4) van Hemert, M. Potential energy surface for the study of inelastic collisions between nonrigid CO and H_2. *The Journal of Chemical Physics* **1983**, *78*, 2345–2354.

(5) Ester, M.; Kriegel, H.-P.; Sander, J.; Xu, X., et al. A density-based algorithm for discovering clusters in large spatial databases with noise. 1996; pp 226–231.
